# Dietary Nutrients, Gut Microbiota, and Cardiac Function: From Metabolic Mechanisms to Clinical Applications

**DOI:** 10.3390/nu18030467

**Published:** 2026-01-31

**Authors:** Lucia Scisciola, Manuela Giovanna Basilicata, Marta Belmonte, Ada Pesapane, Rosaria Anna Fontanella, Nunzia Balzano, Alberta Maria Maddalena Palazzo, Rashmi Joshi, Asad Zia, Giovanni Tortorella, Zeeshan Ulfat, Maryam Arshad, Giuseppe Paolisso

**Affiliations:** 1 Department of Advanced Medical and Surgical Sciences, University of Campania “Luigi Vanvitelli”, P.zza L. Miraglia, 2, 80138 Naples, Italy; manuelagiovanna.basilicata@unicampania.it (M.G.B.); ada.pesapane@unicampania.it (A.P.); rosariaanna.fontanella@unicampania.it (R.A.F.); nunzia.balzano@unicampania.it (N.B.); albertamariamaddalena.palazzo@unicampania.it (A.M.M.P.); rashmi.joshi@unicampania.it (R.J.); asad.zia@unicampania.it (A.Z.); giovanni.tortorella@unicampania.it (G.T.); zeeshan.ulfat@unicampania.it (Z.U.); maryam.arshad@unicampania.it (M.A.); giuseppe.paolisso@unicampania.it (G.P.); 2 Cardiology Unit, Sant’Andrea University Hospital, 00189 Rome, Italy; belmontemarta1@gmail.com; 3 Department of Clinical and Molecular Medicine, Sapienza University of Rome, 00161 Rome, Italy; 4 UniCamillus, International Medical University, 00131 Rome, Italy

**Keywords:** gut microbiota, cardiovascular diseases, microbial metabolites

## Abstract

Background: The heart depends on a continuous and flexible energy supply from fatty acids, glucose, and other substrates. Emerging evidence shows that gut microbiota-derived metabolites—such as trimethylamine-N-oxide (TMAO), short-chain fatty acids (SCFAs), secondary bile acids, indoles, phenylacetylglutamine (PAGln), and branched-chain amino acids—modulate cardiac metabolism and function. Although clinical evidence linking these metabolites to cardiovascular outcomes is expanding, most data remain associative, with limited causal or interventional proof. Methods: A comprehensive narrative review was conducted (PubMed 2010–2025) to integrate preclinical, clinical, and Mendelian randomization studies on microbiota-derived metabolites and cardiovascular disease, complemented by evidence from dietary and interventional trials. Results: Gut-derived metabolites regulate mitochondrial energetics, inflammatory, immune system, and oxidative pathways, and endothelial and platelet activation. Elevated plasma TMAO and PAGln levels are often associated with adverse cardiovascular outcomes, while SCFAs and indole derivatives may related to protective effects. However, findings across cohorts remain heterogeneous, largely due to differences in diet, renal function, and analytical methods. Dietary patterns rich in fiber and plant-based nutrients favor beneficial metabolite profiles, underscoring the nutritional modulation of the gut–heart axis. Conclusions: The diet–microbiota–metabolite axis represents an emerging pathway connecting nutrition to cardiovascular health. Translating this knowledge into prevention and therapy will require large-scale randomized studies and integrated multi-omics approaches. Dietary modulation of microbial metabolism may ultimately become a novel strategy for cardiometabolic protection.

## 1. Introduction

The heart is a metabolically flexible organ, capable of adapting to different energy substrates—fatty acids, glucose, lactate, ketone bodies, and amino acids—to sustain continuous contraction and function. This metabolic flexibility is crucial for maintaining cardiac homeostasis under both physiological and pathological conditions [1]. In recent years, an additional player has emerged in this complex scenario: the gut microbiota, now regarded as a “metabolic organ” capable of transforming dietary nutrients into a wide range of bioactive metabolites with systemic effects [2,3].

The concept of a gut–heart axis has gained ground through studies linking an imbalance in microbial composition (dysbiosis) and gut-derived metabolites to the progression of cardiovascular diseases, including atherosclerosis, hypertension, arrhythmias, and, particularly, heart failure [4,5]. Within this context, several classes of nutrient-derived microbial metabolites have been identified as key mediators. The most studied nutrient-derived microbial metabolites include: (i) Trimethylamine-N-oxide (TMAO), derived from choline, carnitine, and lecithin, associated with adverse cardiovascular outcomes in numerous observational studies [6]; (ii) short-chain fatty acids (SCFA), generated from dietary fiber fermentation, with anti-inflammatory and cardio-energetic effects [7]; (iii) secondary bile acids, acting through nuclear and membrane receptors (FXR and TGR5) and influencing lipid metabolism and contractility [8]; (iv) indoles, derived from tryptophan, associated with heterogeneous cardiovascular effects [9]; (v) phenylacetylglutamine (PAGln), linked to increased platelet reactivity and thrombotic risk [10]; and (vi) branched-chain amino acids (BCAAs), whose altered catabolism has been associated with cardiac remodeling and heart failure, particularly with the heart failure with preserved ejection fraction (HFpEF) phenotype [11].

Despite the growing body of evidence, the field still faces significant limitations and controversies. Much of the literature focuses primarily on TMAO, whereas other metabolites remain underexplored or discussed in a fragmented way. Moreover, many available data derive from observational studies reporting simple associations, without clarifying whether these metabolites are true causal mediators or merely risk biomarkers. Findings are sometimes inconsistent: for instance, while several studies link elevated TMAO levels with cardiovascular mortality and heart failure progression, others fail to confirm an independent association after adjustment for renal function and comorbidities [12,13].

Another relevant gap is the limited integration between molecular mechanisms and clinical data. On the one hand, preclinical models have identified specific signaling pathways of nutrient-derived microbial metabolites—such as activation of G-protein coupled receptor 41/43 (GPR41/43), Aryl Hydrocarbon Receptor (AhR), β-2 adrenergic receptor (β-2AR), oxidative stress, and inflammation—while, on the other hand, translating these findings into clinical contexts remains incomplete [14,15]. Similarly, approaches such as Mendelian randomization and multi-omics analyses, which could provide clues about causality, have yielded heterogeneous and poorly systematized results in current reviews [16].

From a translational perspective, therapeutic opportunities are promising but still immature. Dietary interventions, such as the Mediterranean diet or increased fiber intake, are associated with more favorable metabolic profiles [17]. In contrast, probiotics and prebiotics have shown encouraging but preliminary results in small trials [18]. In parallel, novel pharmacological strategies aim to inhibit key microbial enzymes (e.g., CutC/D in TMA production) or modulate specific receptors for SCFA, bile acids, and indoles [19]. However, large-scale randomized trials with robust clinical endpoints are still lacking to consolidate these options into clinical practice.

In light of these considerations, this review is guided by the working hypothesis that diet–microbiota interactions shape metabolite profiles that may influence cardiac metabolism and function. It aims to provide an integrated overview of nutrient-derived microbial metabolites that influence cardiac metabolism and function, combining mechanistic, clinical, and evidence-based data to identify converging mechanisms and persistent uncertainties, and to highlight future directions for translating microbiota-derived metabolic pathways into cardiovascular prevention and therapy.

## 2. Materials and Methods

A comprehensive literature search was conducted in PubMed/MEDLINE from January 2010 to March 2025. Only articles published in English were considered. Given the interdisciplinary nature of this topic and the evolving evidence on the gut–microbiota–heart axis, we adopted a narrative and integrative approach rather than a formal systematic review and therefore considered multiple study designs. We included peer-reviewed articles published in English that investigated (i) dietary nutrients and/or microbiota-targeted interventions (e.g., probiotics and prebiotics), (ii) gut microbiota-derived metabolites (e.g., TMAO, PAGln, secondary bile acids, SCFAs, indole-related metabolites, and amino acids), and (iii) cardiovascular phenotypes/mechanisms (e.g., atherosclerosis, thrombosis, endothelial dysfunction, and heart failure). Eligible study designs included experimental (in vitro/in vivo), observational studies, and clinical trials, as well as high-quality systematic reviews relevant to mechanistic interpretation. We excluded conference abstracts, editorials/letters without original data, case reports, non-English papers, and studies that did not address the gut microbiota–metabolite axis in relation to cardiovascular outcomes.

The primary search combined keywords related to microbiota, nutrient-derived metabolites, and cardiac function using Boolean operators (AND/OR). The search strategy included the following terms: gut microbiota, intestinal microbiota, microbiome, nutrient-derived metabolites, trimethylamine N-oxide, TMAO, short-chain fatty acids, SCFA, bile acids, indoles, tryptophan metabolism, phenylacetylglutamine, PAGln, branched-chain amino acids, BCAA, cardiac metabolism, heart failure, HFpEF, heart failure with reduced ejection fraction (HFrEF), atherosclerosis, cardiovascular disease, diet, nutrition, metabolomics, metabolic pathways, and nutritional interventions.

Two independent reviewers performed the search to enhance completeness and accuracy. In addition, reference lists of selected articles and relevant reviews were manually screened to identify additional eligible publications. No formal quantitative synthesis (meta-analysis) was planned, as the included studies were heterogeneous in design and analytical methods. The review, therefore, emphasizes convergence across mechanistic, translational, and clinical evidence.

## 3. Overview of Nutrient-Derived Microbial Metabolites

A variety of gut microbiota-dependent metabolites derived from dietary substrates have been implicated in cardiovascular physiology and disease. These metabolites act through distinct molecular pathways, influencing inflammation, thrombosis, myocardial energetics, and remodeling (Table 1).

### 3.1. Trimethylamine/Trimethylamine-N-Oxide (TMA/TMAO)

TMAO is a gut microbiota-derived metabolite originating from trimethylamine (TMA), which in turn is produced from dietary nutrients rich in trimethylamine moieties such as choline, phosphatidylcholine, and carnitine, commonly found in red meat, eggs, and fish [20]. Once absorbed, TMA is transported to the liver, where flavin-containing monooxygenase 3 (FMO3) oxidizes it into TMAO [21]. TMAO has been shown to interfere with reverse cholesterol transport, promoting cholesterol deposition in the arterial wall and accelerating atherosclerosis [22]. Mechanistically, elevated circulating TMAO levels contribute to endothelial dysfunction, oxidative stress, inflammatory activation, and platelet hyperreactivity, thereby promoting vascular injury and atherothrombosis [23,24]. Experimental studies have demonstrated that animal models supplemented with TMAO, choline, or carnitine show increased foam cell formation from macrophages and accelerated atherosclerotic plaque development [21]. Furthermore, in diabetic or metabolically impaired mice, circulating TMAO levels are elevated, whereas reducing TMAO production by knocking down the enzyme FMO3 improves insulin tolerance and reduces cardiovascular issues such as atherosclerosis [25].

### 3.2. Phenylacetylglutamine (PAGln)

PAGln is a gut microbiota-dependent metabolite generated from the microbial metabolism of dietary phenylalanine, followed by host conjugation with glutamine [26]. The gut microbiome is essential for its production, as antibiotic treatment in both mice and humans leads to a transient decline in circulating PAGln levels, confirming its microbial origin [26,27].

PAGln may also modestly influence thrombotic risk by engaging platelet adrenergic receptors and facilitating Ca^2+^-dependent activation, an effect shown to be reversible with carvedilol, suggesting a role for adrenergic signaling in its platelet-related actions [28].

### 3.3. Secondary Bile Acids

Secondary bile acids (BAs) are synthesized in the liver from cholesterol to form primary bile acids, which are secreted into the small intestine to facilitate the absorption and digestion of lipids and fat-soluble vitamins [29]. Within the intestinal lumen, the gut microbiota plays a crucial role in the biotransformation of bile acids [30,31]. This microbial conversion substantially alters the composition and pool size of circulating bile acids, influencing their signaling potential and enterohepatic circulation [30]. Before re-entering the liver via the enterohepatic recirculation, bile acids may undergo additional microbial modifications that affect their receptor affinity and systemic effects [30]. Beyond their digestive functions, bile acids are key signaling molecules that interact with a broad spectrum of receptors, including nuclear receptors such as the farnesoid X receptor (FXR), pregnane X receptor (PXR), and vitamin D receptor (VDR), as well as membrane receptors like the Takeda G-protein-coupled bile acid receptor 1 (TGR5/GP-BAR1) and muscarinic receptors [30,32,33,34,35,36]. FXR and TGR5 are expressed in endothelial cells, vascular smooth muscle cells, and cardiomyocytes, where they influence cholesterol and glucose metabolism, vascular tone, and inflammatory responses [37,38,39,40]. At physiological levels, bile acid-mediated activation of FXR and TGR5 tends to be vasculoprotective, promoting NO-dependent vasodilation, improving endothelial function, and exerting anti-inflammatory and lipid-lowering effects through mechanisms that include NO synthesis, Ca^2+^-dependent K^+^ channel activation, and muscarinic receptor stimulation [40,41,42,43,44]. In contrast, excess secondary bile acids such as deoxycholic acid can cause oxidative injury, mitochondrial dysfunction, endothelial damage, and reduced cardiomyocyte contractility, highlighting their dose-dependent, dual nature [45,46]. Overall, despite the complexity arising from their chemical heterogeneity and receptor promiscuity, current evidence supports bile acids as key modulators of vascular tone, metabolic homeostasis, and inflammation at the intersection of hepatic metabolism, gut microbiota activity, and cardiovascular function [47].

### 3.4. Amino Acids

Both host and microbial enzymes metabolize dietary branched-chain amino acids (leucine, isoleucine, and valine—BCAA). Impaired BCAA catabolism has been implicated in metabolic remodeling of the failing heart, particularly in HFpEF [48]. The accumulation of BCAAs and their toxic intermediates activates mTOR signaling, contributing to myocardial hypertrophy, fibrosis, and impaired energetics [49].

### 3.5. Short-Chain Fatty Acids (SCFA)

Short-chain fatty acids (SCFAs)—primarily acetate, propionate, and butyrate—are produced in the colon through anaerobic fermentation of indigestible dietary fibers such as resistant starch, pectin, inulin, and other complex carbohydrates [50,51]. After absorption, propionate and acetate undergo hepatic metabolism and enter systemic circulation, where they modulate cardiovascular function [52]. SCFAs exert systemic effects via activation of G-protein-coupled receptors (GPR41/FFAR3 and GPR43/FFAR2) expressed on endothelial and immune cells and adipose tissues, and through inhibition of histone deacetylases (HDACs), thereby influencing chromatin remodeling and gene expression [53,54]. Through these mechanisms, they enhance gut barrier integrity, regulate lipid and glucose homeostasis, and reduce systemic inflammation [53,55,56,57]. Experimental evidence indicates that SCFAs ameliorate endothelial dysfunction, lower blood pressure, and attenuate cardiac hypertrophy and fibrosis [58,59,60,61]. Notably, butyrate has been shown to increase HDL levels and reduce LDL concentrations, supporting cardiovascular protection through improved lipid metabolism and reduced oxidative stress [62,63].

### 3.6. Indoles

Indole derivatives, such as indole-3-propionic acid (IPA), are produced from dietary tryptophan by specific gut bacteria. IPA has been shown to exert antioxidant and anti-inflammatory effects, partly by activating the AhR and modulating NAD metabolism [64].

## 4. Mechanistic Pathways Linking Nutrient-Derived Metabolites to Cardiac Function

**Figure 1:**
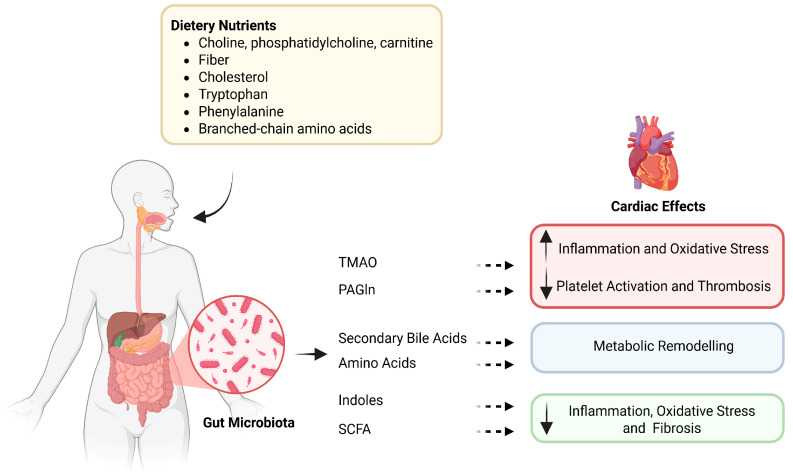
The gut–heart axis: microbial metabolites linking diet to cardiovascular effects. Dietary precursors such as choline, bile acids, and amino acids are metabolized by gut microbes into bioactive compounds, including TMAO, PAGln, SCFAs, and indoles. These metabolites exert diverse effects on the cardiovascular system, modulating inflammation, oxidative stress, thrombosis, and metabolic remodeling. TMAO—Trimethylamine N-oxide; PAGln—Phenylacetylglutamine; SCFAs—Short-chain fatty acids. Dietary nutrients and microbiota-targeted interventions (probiotics, prebiotics, and microbiota-directed drugs) shape gut microbiota composition and the production of specific metabolites, including trimethylamine N-oxide (TMAO), phenylacetylglutamine (PAGln), bile acids, short-chain fatty acids (SCFAs), amino acids, and indole derivatives. These metabolites are differentially associated with cardiovascular diseases: TMAO, PAGln, bile acids, and amino acids are linked to atherosclerosis, heart failure, and major adverse cardiovascular events (MACE), whereas SCFAs and some indole derivatives are often associated with protective phenotypes; however, these effects are context-dependent and may vary by metabolite species and host conditions. This schematic summarizes reported associations and proposed mechanisms; arrows do not imply established causality in humans.

Gut-derived metabolites influence cardiac physiology through a range of molecular and cellular mechanisms (Figure 1). These include modulation of myocardial energetics, inflammatory and oxidative stress signaling, endothelial and platelet function, and structural remodeling.

### 4.1. Inflammation and Immune Signaling

Low-grade chronic inflammation is a central mechanism linking gut-derived metabolites to cardiovascular disease. Among these, TMAO has been associated with pro-inflammatory signaling, and experimental studies suggest it may amplify immune and vascular responses. Elevated plasma TMAO levels have been associated with enhanced NF-κB signaling activation, increased interleukin production (IL-1β and IL-6), and upregulation of adhesion molecules such as Vascular Cell Adhesion Molecule-1 (VCAM-1) and Intercellular Adhesion Molecule-1 (ICAM-1) in endothelial cells. It has also been observed that TMAO primes the NLRP3 inflammasome, thereby contributing to endothelial injury and immune cell infiltration [22,65]. At the same time, TMAO has been reported to promote reactive oxygen species (ROS) generation by stimulating Nicotinamide Adenine Dinucleotide Phosphate (NADPH) oxidase activity and by reducing mitochondrial antioxidant defenses, including Sirtuin 1/3 (SIRT1/3), and superoxide dismutase (SOD2). This imbalance is correlated with reduced NO bioavailability and endothelial dysfunction [22,66,67]. Conversely, SCFAs and indole derivatives exhibit anti-inflammatory and antioxidant properties [68]. Butyrate and propionate have been shown to inhibit HDACs, restore the Treg/Th17 balance, and suppress NF-κB-dependent cytokine expression, while also upregulating antioxidant enzymes such as catalase and glutathione peroxidase [69].

Similarly, IPA has been associated with reduced oxidative stress and improved endothelial integrity by activating the AhR [70,71].

Under dysbiotic conditions, however, tryptophan metabolism shifts toward the production of kynurenine and indoxyl sulfate. These metabolites have been reported to induce oxidative stress and endothelial apoptosis via AhR–p38 MAPK activation, thereby compromising vascular integrity [72,73].

Activation of FXR (and, in some contexts, TGR5) by specific bile acid species has been associated with anti-inflammatory signaling in experimental models [74]. However, bile acid effects are receptor-, species-, and concentration-dependent, and excessive secondary bile acid accumulation has been linked to oxidative injury and endothelial dysfunction [75]. Collectively, these findings underscore the context-dependent, pro- versus anti-inflammatory actions of bile acids in the cardiovascular system [76].

### 4.2. Oxidative Stress

Oxidative stress is a major contributor to cardiovascular pathology within the gut–heart axis. TMAO has been reported to increase ROS formation by stimulating NADPH oxidase and suppressing mitochondrial antioxidant enzymes such as SOD2 through inhibition of sirtuins (SIRT1 and SIRT3) [22,66,67]. These changes may contribute to mitochondrial dysfunction, endothelial activation, and diminished NO bioavailability, thereby accelerating vascular injury.

Conversely, SCFAs and indole derivatives exhibit robust antioxidant activity [76]. Butyrate enhances the expression of antioxidant enzymes such as catalase and glutathione peroxidase [77], while IPA functions as a potent free radical scavenger, protecting cardiomyocytes from lipid peroxidation [71,78]. In addition, bile acid signaling via FXR and TGR5 modulates redox balance. Actually, FXR activation attenuates ET-1-mediated vasoconstriction via AMPK signaling [79], whereas TGR5 activation enhances cAMP-mediated antioxidant responses [39].

In preclinical models, chronic TMAO exposure exacerbates oxidative stress and accelerates atherosclerosis [22]. FXR activation by secondary bile acids is linked to reduced oxidative stress, in part through their lipid-lowering and anti-inflammatory actions, which improve endothelial function [79,80]. In contrast, high concentrations of secondary bile acids, such as deoxycholic acid, can trigger oxidative injury and mitochondrial dysfunction, thereby promoting vascular damage [39,81]. These observations highlight that bile acids may either limit or exacerbate oxidative stress depending on receptor engagement, molecular species, and concentration [82].

### 4.3. Endothelial and Platelet Function

The endothelium serves as a dynamic interface between the circulation and the tissue, regulating vascular tone, permeability, and hemostasis. Gut-derived metabolites influence endothelial and platelet function in both protective and pathological directions. TMAO and PAGln have been shown in experimental settings to increase platelet responsiveness by activating adrenergic receptors (α2A, α2B, β1, and β2), suggesting an increased intracellular calcium flux and thrombus formation [83]. This pro-thrombotic state is reinforced by oxidative stress, which promotes leukocyte adhesion and endothelial activation [83,84].

TMAO suppresses sirtuin activity and endothelial nitric oxide synthase (eNOS) function, leading to reduce NO bioavailability and impaired vasodilation [85]. PAGln-mediated adrenergic signaling further enhances NF-κB-dependent inflammatory gene expression, upregulating VCAM-1 and ICAM-1 [86]. Mechanistically, PAGln has been shown to modulate platelet function and thrombosis risk. It activates the adrenergic receptors α_2_A, α_2_B, and β_2_ expressed on human platelets, thereby enhancing intracellular Ca^2+^ signaling and lowering the activation threshold for classical platelet agonists such as ADP [28]. This receptor-mediated mechanism can promote platelet hyperresponsiveness and collagen adhesion under flow conditions, thereby increasing thrombotic potential [28]. In experimental models, carotid arterial thrombosis induced by PAGln was reversed by carvedilol, a non-selective adrenergic receptor antagonist, demonstrating that the pro-thrombotic effects of PAGln are mediated via adrenergic signaling [28]. These processes may create a sustained inflammatory and thrombogenic milieu conducive to atherosclerotic plaque progression.

By contrast, SCFAs strengthen endothelial integrity and enhance eNOS-mediated NO production [58]. Butyrate promotes vasodilation and barrier function, while propionate modulates sympathetic activity via GPR41, thereby reducing vascular tone and blood pressure [69,87]. Bile acids also contribute to vascular homeostasis, acting on FXR and TGR5. In particular, within the vasculature, both receptors contribute to the regulation of vascular tone and endothelial homeostasis [88]. TGR5 activation promotes vasodilation via NO signaling, whereas FXR stimulation improves endothelial function and reduces inflammation [89,90]. The vasodilatory effects of bile acids are mediated by multiple mechanisms, including enhanced NO synthesis, activation of Ca^2+^-dependent K^+^ channels, and stimulation of muscarinic receptors [90,91,92]. At supraphysiological concentrations, however, bile acids can impair endothelial integrity and alter vascular relaxation, again emphasizing their dose-dependent dual nature [8].

### 4.4. Myocardial Energetics

The myocardium is one of the most metabolically active tissues in the body, requiring a continuous supply of ATP to sustain contraction and relaxation. Under physiological conditions, fatty acid oxidation provides approximately 60–70% of cardiac ATP production, with glucose and lactate serving as complementary energy substrates. However, during metabolic stress, SCFAs may contribute as alternative substrates that can enhance mitochondrial efficiency and preserve cardiac energetics [63]. SCFAs—principally acetate, propionate, and butyrate—exert effects through the activation of GPR41 and GPR43 and inhibition of histone deacetylases (HDACs) [77,93]. Butyrate promotes mitochondrial biogenesis and oxidative phosphorylation by activating Peroxisome Proliferator-Activated Receptor Gamma Coactivator (PGC)-1α and AMP-activated protein kinase (AMPK), thereby improving mitochondrial efficiency and energy conservation [94]. Propionate sustains anaplerotic flux into the tricarboxylic acid (TCA) cycle, preserving ATP synthesis under hypoxic or nutrient-limited conditions. Conversely, impaired metabolism of BCAAs (leucine, isoleucine, and valine) leads to accumulation of branched-chain ketoacids (BCKAs), which interfere with mitochondrial oxidative capacity and activate mechanistic Target Of Rapamycin Complex 1 (mTORC1) signaling. This maladaptive activation promotes protein synthesis, cardiomyocyte hypertrophy, and diastolic dysfunction, key features of heart failure with HFpEF [95,96]. In addition, bile acids influence cardiomyocyte function, in part by affecting mitochondrial integrity. Excessive accumulation of secondary bile acids, such as deoxycholic acid, can induce mitochondrial dysfunction, oxidative injury, and endothelial damage, thereby contributing to vascular and myocardial pathology [97]. At higher concentrations, bile acids have also been reported to reduce cardiomyocyte contractility in a dose-dependent manner, indicating a potential detrimental impact on myocardial performance when levels exceed the physiological range [98].

Thus, cardiac energy metabolism is a critical node through which nutrient-derived metabolites influence myocardial performance and metabolic flexibility.

### 4.5. Cardiac Remodeling and Fibrosis

Cardiac remodeling represents the culmination of metabolic, inflammatory, and oxidative stress-related insults driven by gut-derived metabolites. TMAO promotes fibroblast activation and collagen synthesis via TGF-β/Smad signaling, thereby contributing to interstitial fibrosis and impaired ventricular compliance [99]. Chronic TMAO exposure also exacerbates hypertrophy and stiffness, mimicking clinical features of HFpEF.

Likewise, excessive BCAA accumulation triggers mTORC1–S6K1–4EBP1 signaling, stimulating protein synthesis and hypertrophic remodeling [100]. Impaired BCAA catabolism in macrophages contributes to increased pro-inflammatory cytokine release, establishing a vicious cycle between metabolic stress and inflammation.

Conversely, SCFAs and indole derivatives counteract fibrosis by inhibiting TGF-β-driven myofibroblast differentiation and extracellular matrix deposition [63]. Bile acid receptors FXR and TGR5 regulate matrix turnover by suppressing fibrotic gene expression and improving mitochondrial quality control [101]. Restoring the balance of these metabolites can therefore mitigate pathological remodeling and preserve myocardial architecture.

### 4.6. Electrophysiological Effects

Emerging data indicate that gut-derived metabolites also modulate cardiac electrophysiology. Secondary bile acids affect ion channel function—particularly calcium and potassium channels—through FXR/TGR5-dependent mechanisms, altering action potential duration and predisposing to arrhythmias [101]. PAGln-driven adrenergic stimulation further enhances calcium influx and delays repolarization, heightening arrhythmogenic potential [102]. Moreover, accumulation of BCAAs and kynurenine pathway metabolites disrupts mitochondrial potential and elevates ROS production in cardiomyocytes, contributing to electrical instability and arrhythmogenesis [72,96]. These findings highlight the intricate interplay between metabolic and electrophysiological signaling in the gut–heart axis.

## 5. Clinical Evidence

Clinical research investigating gut-derived, nutrient-dependent metabolites in cardiovascular disease has expanded rapidly in recent years. Nevertheless, methodological limitations and confounding factors still hinder causal interpretation and clinical translation. Current knowledge mainly derives from observational and case–control studies, while randomized interventional evidence remains limited.

### 5.1. Heart Failure (HFpEF vs. HFrEF): Differences in Metabolite Profiles

Altered gut microbiota composition and distinct metabolomic signatures have been reported in patients with heart failure, with consistent depletion of SCFA-producing taxa and enrichment of pathobionts such as Proteobacteria [103]. Circulating TMAO levels are frequently elevated in both HFpEF and HFrEF and have been associated with adverse prognosis [104], although adjustment for renal function often attenuates these associations [105,106]. While some Mendelian randomization (MR) studies suggest a modest causal contribution of TMAO to heart failure or coronary artery disease [107], others fail to confirm these findings after accounting for pleiotropy and population differences. Conversely, decreased SCFA concentrations and impaired BCAA catabolism have been reported primarily in HFpEF [100,108,109,110], supporting a link between metabolic remodeling and diastolic dysfunction. In addition, preclinical studies indicate that IPA protects against diastolic dysfunction in models of HFpEF and mitigates sepsis-induced cardiac dysfunction [9].

### 5.2. Atherosclerosis, Acute Myocardial Infarction, and Stroke: Prospective Associations (TMAO and PAGln)

Prospective cohort studies and meta-analyses have reported positive associations between higher plasma concentrations of TMAO or PAGln and the incidence of major adverse cardiovascular events (MACE), including myocardial infarction, stroke, and cardiovascular mortality [111,112,113]. Actually, in a large cohort of patients undergoing elective coronary angiography, plasma TMAO levels were independent predictors of stroke, myocardial infarction, revascularization, and death [114]. PAGln, in particular, demonstrates a mechanistic connection with platelet adrenergic signaling [27,28], supporting the biological plausibility of a link to thrombosis. However, the strength and consistency of these associations vary considerably across studies, largely due to differences in population size, analytical platforms, and the adjustment for dietary and metabolic confounders [115,116,117,118].

## 6. Emerging Biomarkers: Robust vs. Controversial

Among the diverse metabolites produced by the gut microbiota, several have been proposed as potential biomarkers of cardiovascular disease, though the strength and consistency of the supporting evidence vary widely. Because elevated plasma TMAO levels have been linked to increased risk of all-cause mortality and major cardiovascular events across multiple cohorts and meta-analyses [21,119,120], TMAO could be considered a risk-stratification tool in both primary and secondary prevention settings. A second emerging biomarker is PAGln, a phenylalanine-derived metabolite associated with heightened thrombotic risk. Elevated PAGln levels have been observed in patients with heart failure and have been linked to increased platelet reactivity and adverse clinical outcomes [10,28,121]. In contrast, other metabolites, such as SCFAs, present a more complex and context-dependent picture. Butyrate appears elevated in HFrEF but reduced in HFpEF, suggesting phenotype-specific metabolic adaptations [7]. These inconsistencies highlight the need for standardized measurement methods and better-controlled clinical studies.

Indole derivatives also show promise. Preclinical data suggest anti-inflammatory and antioxidant effects, and early clinical observations report lower IPA levels in patients with HFpEF compared to healthy controls [9]. However, the available human evidence remains preliminary, and larger cohorts are required to confirm their diagnostic and prognostic utility. Similarly, BCAAs and their metabolic byproducts have been implicated in the pathophysiology of heart failure, particularly HFpEF. Impaired BCAA catabolism may contribute to myocardial metabolic dysfunction, and elevated circulating levels have been observed in both HFrEF and HFpEF [108,122]. Nonetheless, these findings are primarily associative, and it remains unclear whether BCAA alterations are causal or merely reflect systemic metabolic dysregulation. Finally, specific metabolites—despite extensive investigation—remain the subject of debate. This is particularly true for TMAO, whose causal role is still uncertain. While strong associations with cardiovascular outcomes have been repeatedly observed, Mendelian randomization and multi-omics studies have produced conflicting results, failing to confirm a direct pathogenic role in many cases [21,107,119,120,123]. These discrepancies raise the possibility that TMAO may serve more as a marker of dysbiosis or renal dysfunction than as an active disease mediator. In conclusion, while TMAO and PAGln currently stand out for their reproducibility and translational potential, other metabolites such as SCFAs, indoles, and BCAAs offer intriguing, albeit less established, avenues for biomarker discovery. Future research will need to integrate clinical cohorts, mechanistic insights, and interventional studies to clarify their roles and define their place in cardiovascular risk stratification and prevention.

## 7. Dietary Modulation and Interventional Perspectives

Dietary patterns are a primary determinant of gut microbial composition and the profile of microbial-derived metabolites. Mediterranean and plant-based dietary regimens—characterized by high intake of fiber, polyphenols, and omega-3 fatty acids—are associated with enhanced production of SCFAs and favorable modulation of bile acid signaling pathways. These effects contribute to improvements in lipid metabolism, endothelial function, and anti-inflammatory status [50,124,125,126,127]. High-fiber intake specifically augments colonic fermentation, resulting in increased systemic levels of acetate and butyrate, which, in turn, support mitochondrial biogenesis, oxidative phosphorylation, and the suppression of pro-inflammatory cytokine production [124,128,129].

Microbiota-targeted nutritional interventions, including probiotics and prebiotics, represent pragmatic adjunct strategies to modulate gut-derived metabolites implicated in CVD. Although available evidence suggests modest improvements in selected cardiometabolic risk markers (e.g., blood pressure, lipid and glycemic profiles, and inflammatory indices), results vary substantially depending on formulation, dose, and the population studied [130]. Mechanistically, these approaches may promote SCFA-producing taxa and reshape bile acid pools, potentially influencing downstream FXR/TGR5 signaling [131]. Conversely, interventions aimed at modulating the choline/carnitine-derived TMA/TMAO pathway have yielded mixed results; in some controlled trials, probiotic supplementation was not associated with consistent reductions in circulating TMAO levels under fasting conditions or following a dietary challenge [132,133]. Similarly, inulin supplementation did not reduce plasma TMAO concentrations in adults at cardiometabolic risk [134]. Beyond metabolite modulation, probiotic and prebiotic supplementation—particularly formulations containing Lactobacillus and Bifidobacterium strains—has been associated with reduced systemic inflammatory markers and improved left ventricular function in preliminary clinical studies involving heart failure patients [135,136]; however, these findings are primarily derived from small-scale or pilot trials, and larger randomized controlled studies are required to confirm therapeutic efficacy and generalizability.

Dietary restriction of red meat and choline-rich foods (e.g., egg yolks and liver) has been proposed as a strategy to reduce circulating levels of TMA, a pro-atherothrombotic metabolite produced through gut microbial metabolism of dietary trimethylamine-containing compounds [20,137]. In parallel, pharmacological inhibition of microbial TMA production via targeting of CutC/D enzymes offers a novel therapeutic avenue, as evidenced by preclinical studies demonstrating reduced TMAO levels and attenuated atherosclerosis [138,139].

Collectively, available evidence suggests that nutritional modulation of gut-derived metabolites may represent a potential adjunct approach for cardiovascular prevention. However, most human data remain associative and interventional findings are heterogeneous; therefore, these strategies should be considered hypothesis-generating until confirmed in adequately powered randomized controlled trials with robust clinical endpoints. Targeting the diet–microbiota–metabolite axis may thus complement conventional cardiometabolic therapies by addressing upstream metabolic and inflammatory pathways.

## 8. Conclusions

The growing understanding of the diet–microbiota–heart axis reveals a complex network of interactions that shape cardiovascular health. Microbial metabolites such as TMAO and PAGln have shown reproducible associations with adverse cardiac outcomes and mechanistic links to inflammation, thrombosis, and remodeling. SCFAs, selected indole derivatives, and bile acid signaling have been associated with potentially beneficial effects on metabolic flexibility, endothelial function, and redox balance, although these relationships are context- and concentration-dependent. Nevertheless, the clinical relevance of many of these metabolites remains limited by methodological heterogeneity, confounding factors (e.g., renal function), and inconsistent evidence from Mendelian randomization or interventional trials.

Dietary patterns emerge as key upstream modulators of these microbial pathways. High-fiber and plant-based diets support beneficial microbial activity and metabolite production, while targeted interventions—such as probiotics, prebiotics, and enzyme inhibition—represent promising but still exploratory therapeutic strategies.

To translate this knowledge into clinical practice, future research must address current gaps through large-scale, longitudinal, and mechanistically informed studies. Integrating multi-omics profiling, microbial functional assays, and randomized interventions will be essential to move beyond correlation and toward causally grounded, personalized approaches for cardiovascular prevention and therapy.

## Figures and Tables

**Table 1:** Major nutrient-derived microbial metabolites and their cardiovascular relevance.

Metabolite	Dietary Source	Microbial Pathway	Molecular Targets	Cardiac Effects
TMA/TMAO	Choline, phosphatidylcholine, carnitine	Hepatic FMO3	Endothelium, platelets, ROS pathways	↑ inflammation, ↑ thrombosis, adverse prognosis in HF
PAGln	Phenylalanine	Microbial catabolism + host conjugation	Adrenergic receptors (α2A, α2B, β2)	↑ platelet reactivity, ↑ thrombosis risk
Secondary Bile Acids	Primary bile acids (host liver)	Bacterial deconjugation, dehydroxylation	FXR, TGR5, PXR, VDR, and muscarinic receptors	Modulation of lipid metabolism, vascular tone, contractility, and inflammation
Amino Acids	Protein-rich foods	Host/microbial metabolism, impaired catabolism	mTOR, mitochondrial pathways	Hypertrophy, fibrosis, impaired energetics (esp. HFpEF)
SCFA (acetate, propionate, butyrate)	Dietary fiber, resistant starch	Anaerobic bacterial fermentation	GPR41/43, HDAC inhibition	↑ mitochondrial energetics, anti-inflammatory, anti-fibrotic
Indoles (e.g., IPA)	Tryptophan	Bacterial tryptophan metabolism	AhR, NAD pathways	Antioxidant protects against diastolic dysfunction

↑ denotes an increase. TMA/TMAO: (Trimethylamine N-oxide); FMO3: (Flavin-containing monooxygenase 3); ROS (Reactive Oxygen Species); HF (Heart Failure); SCFA: Short-Chain Fatty Acid; GPR41/43: G-protein coupled receptor 41 and 43; HDAC: Histone Deacetylase; FXR: Farnesoid X receptor; TGR5: Takeda G protein-coupled receptor 5; PXR: Pregnane X Receptor; VDR: Vitamin D receptor; IPA: Indole 3-propionic acid; AhR: Aryl hydrocarbon receptor; NAD: Nicotinamide Adenine Dinucleotide; α2A: alpha-2A adrenergic receptor; α2B: alpha-2B adrenergic receptor; β2: beta-2 adrenergic receptors; mTOR: mechanistic target of rapamycin; HFpEF: Heart Failure with Preserved Ejection Fraction.

## Data Availability

Not applicable.

## Abbreviations

The following abbreviations are used in this manuscript:

AhR	Aryl hydrocarbon receptor
AMPK	AMP-activated protein kinase
ATP	Adenosine triphosphate
BA	Bile acid
BCAA	Branched-chain amino acid
BCKA	Branched-chain keto acid
CKD	Chronic kidney disease
cAMP	Cyclic adenosine monophosphate
CVD	Cardiovascular disease
eNOS	Endothelial nitric oxide synthase
ET-1	Endothelin-1
FMO3	Flavin-containing monooxygenase 3
FXR	Farnesoid X receptor
GPR41	G protein-coupled receptor 41 (free fatty acid receptor 3, FFAR3)
GPR43	G protein-coupled receptor 43 (free fatty acid receptor 2, FFAR2)
HDAC	Histone deacetylase
HF	Heart failure
HFpEF	Heart failure with preserved ejection fraction
HFrEF	Heart failure with reduced ejection fraction
ICAM-1	Intercellular adhesion molecule-1
IL-1β	Interleukin-1 beta
IL-6	Interleukin-6
IPA	Indole-3-propionic acid
MACE	Major adverse cardiovascular events
MR	Mendelian randomization
mTOR	Mechanistic target of rapamycin
mTORC1	Mechanistic target of rapamycin complex 1
NAD	Nicotinamide adenine dinucleotide
NADPH	Nicotinamide adenine dinucleotide phosphate
NF-κB	Nuclear factor kappa-light-chain-enhancer of activated B cells
NO	Nitric oxide
NLRP3	NOD-like receptor family pyrin domain-containing 3
PAGln	Phenylacetylglutamine
PCI	Percutaneous coronary intervention
PGC-1α	Peroxisome proliferator-activated receptor gamma coactivator-1 alpha
PXR	Pregnane X receptor
ROS	Reactive oxygen species
SCFA	Short-chain fatty acid
SIRT1	Sirtuin 1
SIRT3	Sirtuin 3
SOD2	Superoxide dismutase 2
TCA	Tricarboxylic acid cycle
TGF-β	Transforming growth factor beta
TGR5	Takeda G protein-coupled receptor 5 (G protein-coupled bile acid receptor 1)
TMA	Trimethylamine
TMAO	Trimethylamine N-oxide
VCAM-1	Vascular cell adhesion molecule-1
VDR	Vitamin D receptor
